# Restrictive Reciprocal Obligations: Perceptions of Parental Role in Career Choices of Sub-Saharan African Migrant Youths

**DOI:** 10.3389/fpsyg.2021.576193

**Published:** 2021-07-09

**Authors:** Peter Akosah-Twumasi, Theophilus I. Emeto, Daniel Lindsay, Komla Tsey, Bunmi S. Malau-Aduli

**Affiliations:** ^1^College of Medicine and Dentistry, James Cook University, Townsville, QLD, Australia; ^2^College of Public Health, Medical and Veterinary Sciences, James Cook University, Townsville, QLD, Australia; ^3^College of Arts, Society and Education, James Cook University, Cairns, QLD, Australia

**Keywords:** sub-Saharan Africa, migrants, youths, career choice, family needs, parental expectations

## Abstract

This study employed interpretivist, grounded theory method and utilized semi-structured interviews to explore how 31 African migrant high school and university students from eight sub-Saharan African representative countries and currently residing in Townsville, Australia, perceived the roles of their parents in their career development. The study findings revealed that the support (financial, social and emotional) and encouragement (sacrificial love, role modeling and guidance) received from parents underpinned the youths’ perceptions of their parents as influential in their career trajectories. Though participants acknowledged their indebtedness to parents and the system that nurtured them, they faced a dilemma conforming to parental preference or personal conviction, which presented “a fork in the career decision-making road.” Study findings indicate that participants’ reactions and strategies for negotiating parental approval differ based on entry status and gender. Most participants, particularly those with professional entry status, conformed to their parents’ career choice for fear of failure, while a few who followed their personal interests negotiated parental approval through dialogue and educating parents. Male participants with humanitarian entry status opposed their parents’ career preferences and followed their own personal interests. Taken together, all participants had strong desire to obtain parental approval and whether sought early or later, the main focus for all participants was prioritizing family needs and obligations. The practical implications of these findings for all stakeholders are discussed.

## Introduction

Choosing a career path is challenging for youths as they explore employment options that match their abilities and interests. In most individualistic societies, freedom and autonomy are promoted, allowing independent choice of career and work, often with parental support (Taylor and Wilson, 2012; Sovet and Metz, 2014). In contrast, group interests tend to take precedence over the individual’s interests in collectivist societies (Fouad et al., 2008; Shea et al., 2009). The views and concerns of family, friends and significant others in collectivist societies are essential when choosing a career path; therefore, youths often depend on them for guidance (Gelfand and Christakopoulou, 1999; Whiston and Keller, 2004). Socio-economic context and labour market are particularly important because of their influence on job availability in these settings (Metz et al., 2009). Studies have shown that career decisions are influenced by job markets, particularly in collectivist societies (Ryan and Deci, 2000; Edwards and Quinter, 2011; Bakar et al., 2014). Youths are constrained in their career aspirations and choices by prevailing job market trends, such as salary, prestigious jobs, promotion opportunities and job accessibility (Choi and Kim, 2013; Jin and Paulsen, 2017; Atitsogbe et al., 2018). These socio-cultural contextual issues imply that fewer career opportunities are available to them, hence the reliance on parental guidance. Youths in collectivist cultures usually do not have the final say about their educational and career decisions due to a societal belief that children do not have the maturity and capacity to make prudent decisions (Howard and Walsh, 2011).

Studies within Asia, Africa, Latin America and portions of rural Europe predominantly identified collective identity, emotional dependence and the importance of family (Singelis et al., 1995; Kim and Atkinson, 2002). In these societies, academic excellence and success in other facets of life bring honor to the family unit (Singaravelu et al., 2005). Furthermore, youths imbibe reciprocity from the family unit as a common feature in most collectivist societies (Kitayama et al., 2007). Multiple studies lend support to youths from collectivist societies perceiving parental expectations as making significant contributions to positive career-related outcomes (Fouad et al., 2008; Sawitri and Creed, 2015; Kim et al., 2016). However, whether the same can be said when these youths migrate with their families to predominantly individualistic societies, like Australia, requires further inquiry.

Cross-cultural migration makes it possible for youths from individualistic and collectivist societies to interact with each other and exposes them to how career pathways are determined in different cultures. This can influence their original world view regarding career decision-making (Dastoor et al., 2005; Gokuladas, 2010). For many migrant youths, choosing a profession is even more complex as they must balance their personal interests with career preferences endorsed by their parents as they integrate into a new culture (Chen et al., 2002; Shen, 2015; Kumar and Pansari, 2016). Most studies on migrant children’s perceptions of parental career related behaviors have been conducted with participants from Asia (Chen et al., 2002; Cheung and Arnold, 2014; Hashim and Embong, 2015; Shen, 2015; Kim et al., 2016; Hui and Lent, 2018). However, research on the perceptions of sub-Saharan African (SSA) migrant youths regarding the roles of their parents in their career decision-making is limited (Kumar and Pansari, 2016; Wambu et al., 2017). Forty-eight African countries constitute the SSA region, and this group of people have many cultural and historical similarities, which reflect philosophical affinity and kinship (UNSD, 2019).

Recent Australian Census data suggest that SSA migrants are migrating to Australia at an increased rate (ABS, 2018). The 2016 Census data show that the SSA population in Australia increased from 3,522 in 1986 to 388,683 in 2016, representing about 1.6% of Australia’s total population, and it is among the fastest developing ethnic groups in Australia (ABS, 2018). Given the increasing numbers of families transitioning cross-culturally from SSA countries, it is important to understand the perceptions of SSA migrant youths of their parents’ roles in their career development in Australia. The realization that the national children’s right framework within Australia (Cumming and Mawdsley, 2005) allows children to negotiate the selection of their career path with their parents or even override their parents’ preferences might exacerbate the youths’ already challenging process of career decision-making, particularly if their parents are insistent on maintaining their heritage cultural practices (Akosah-Twumasi et al., 2018, 2020a,b).

When the youth’s career aspirations are compatible with parental expectations, then any guidance from parents is considered as being positive (Otto, 2000). However, if the career interests are different from parental expectations, then parental guidance will be misconstrued as being intrusive and could affect youths emotional wellbeing and academic performance (Laursen and Collins, 2009; Pinquart and Gerke, 2019). In such circumstances, the role of a career counselor is crucial as their intervention can be beneficial for both parents and students (Boerchi and Tagliabue, 2018). Exploration of SSA migrant youths’ perceptions of their parents’ role in their educational and career construction can foster a deeper understanding of the contextual parent–child relational dynamics. Findings of such explorative studies can also serve as a resource or guide for parents, teachers and career counselors in assisting SSA youths with their career support needs. This can ultimately ease any tension that arises between family members and promote family cohesion for smooth integration. This study explored the perceptions of SSA migrant youth living in Townsville, Australia, about their parents’ involvement in their career choices and how they cope with any conflict of interest between their choices of career and the expectations of their parents. The study was guided by the research question: How do SSA migrant youths perceive the roles of their parents in their career decision making?

### Theoretical Framework

This paper draws on and attempts to anchor the study findings on two career development models that have been propounded in the Western literature to explain the career decision-making processes of individuals. These models are the Social Learning Theory of Career Decision Making (SLTCDM) by Krumboltz et al. (1976) and the Social Cognitive Career Theory (SCCT) by Lent et al. (1994). These models were chosen because they offer valuable theoretical perspectives from which career development in contemporary contexts could be examined.

Building on Bandura’s (1971) social learning theory, Krumboltz and his colleagues introduced the social learning theory of career decision-making in 1976. The authors proposed four major categories of career selection influencers, namely genetic or personal characteristics, work environment, learning experience and task skills. These factors act as constraints or facilitators, and the interaction between them results in the development of socio-cognitive beliefs that significantly impact on career development (Mitchell et al., 1979). Krumboltz et al. (1976), p.71 emphasizes that career decision-making is “influenced to a large extent by factors usually outside the control of any one individual”. Similarly, SCCT posits that career decision-making is a function of a reciprocal relationship between three intricately linked variables – self-efficacy beliefs, outcome expectations and goal-setting behavior (Bandura, 1986; Lent et al., 1994). The theory also emphasizes the dominant influence of variables, such as economic need, family pressures or educational limitations, on adolescents’ career decision making processes (Lent et al., 2002).

## Materials and Methods

### Study Context and Participants

SSA migrant youths attending secondary school and university in Townsville, Queensland, Australia, were purposively sampled and recruited to participate in this study. Townsville is one of the fastest growing regions in Queensland, Australia, with a diverse population (ABS, 2018), including SSAs who are mostly from collectivist societies and practice patrilineal and matrilineal kinship with much respect afforded to the hierarchical structures in the family, community and society (UNSD, 2019). In these societies, relatives are considered as brethren with the responsibility of being companions and burden sharers to cater for the needs of all family members especially the most vulnerable ones (children and the elderly; Hofstede, 2001; Alber et al., 2010). As a society with collectivist tendencies, individual concerns receive communal response because maintaining family cohesion, solidarity and unity of purpose is a shared perception among SSA families (Hofstede, 2001).

Participants were SSA migrant youths who had migrated to Australia with their parents on either skilled or humanitarian grounds and were in high school or studying at different levels at tertiary institutions. According to the International Organization for Migration (IOM, 2020), humanitarian migrants are individuals and families in need of urgent protection, including but not limited to refugees, asylum seekers and migrants in vulnerable situations requiring assistance and care. Humanitarian migrants can be either professionals with university degrees or people with limited qualifications. It is not their level of training or work experience that characterizes them as humanitarian migrants, but what causes them to leave their heritage countries. However, irrespective of migrants’ entry statuses, when they upskill themselves and acquire the host country’s qualifications and training, they stand to gain meaningful employment in the host country. According to Crowley-Henry et al. (2018), professional migrants, also referred to as skilled migrants, are people in transit who already possess university degrees or extensive work experience in their professional field at the time they leave their heritage countries to seek employment elsewhere. In most receiving countries, such as Australia, professional migrants are sought after because they either already have a job lined-up or they have an increased likelihood of gaining employment to achieve economic independence (Birrell, 2003). Additionally, professional migrants contribute with their skills, knowledge and expertise to fill skill shortages in the host country’s labour market and help sustain the nation’s economy.

### Recruitment

This study was part of a larger exploratory study that involved SSA migrant parents and their children (Akosah-Twumasi et al., 2020a). A purposive sampling method was used to recruit participants. Study participants included youths of SSA descent and aged between 13 and 29 years. The term “youth” is used in this context to refer to adolescents within the period of transitioning from the dependence of childhood to adulthood independence and awareness of their interdependence as members of a community (UNESCO, 2016). SSA youths whose parents were not residing in Australia and those who were in higher institutions but were older than 29 years were excluded from participating in the study.

### Study Design

The study employed grounded theory (GT) methods to gain in-depth understanding of SSA migrant youths’ perceptions of parental influence on their career choices. Grounded theory represents both a method of inquiry and a resultant product of that inquiry, and it aims to generate theory that is grounded in the data (Bryant and Charmaz, 2007). The authors followed Strauss and Corbin’s (1990) interpretivist method of GT, which utilizes a sociological perspective that relies on the symbolic meaning people ascribe to the processes of social interaction. This approach addresses the subjective meaning people place on objects, behaviors or events based on what they believe is true (Clarke, 2005). James Cook University’s Human Research Ethics Committee granted approval for this study (ethics approval numbers, H7006 and H7374).

### Data Collection

Semi-structured interviews were used as the procedure of inquiry. Data collection *via* semi-structured interviews occurred between August 2017 and September 2018, and interview sessions were held at locations chosen by the participants. PAT facilitated all the interview sessions, and BSMA observed the first interview session to ensure adherence to the interview protocol. Interview questions posed to participants included how they decided on their preferred careers; the support/contribution from parents (family members) towards their career development; participants’ feelings about any divergent views in career matters; participants approach to negotiating competing cultural values; the outcome of the career choice; and past and current relationship with parents (family members). Each interview commenced with a verbal acknowledgement of consent. To avert social desirability bias during the interviews, participants were assured of the researchers’ adherence to confidentiality and anonymity protocols and that there were no right or wrong responses. Additionally, an interview guide, including probing questions and prompts for personal stories, was utilized for the interviews. The interviews continued until data saturation was achieved (Birks and Mills, 2015). For confidentiality purposes, participants were given pseudonyms to maintain anonymity. Involvement in the study was purely voluntary, and there were no incentives, monetary or otherwise, offered to participants. Interview sessions ranged from 30 to 60 min.

### Data Analysis

Participants’ responses were transcribed, and the data were analyzed in Nvivo Software version 11 (Pro, 2016), in line with Strauss and Corbin’s (1990) three phases of GT coding – open, axial and selective, utilizing both inductive and abductive reasoning (Birks and Mills, 2015). In the open coding, the transcribed interviews were examined line-by-line to develop the initial coding for descriptive categories which are then grouped together around commonalities to influence the development of significant subcategories. Axial coding was subsequently carried out to establish the relationships between the developed subcategories and to reconstruct the concepts within them into large and more focused categories. At this stage, the researchers used a coding paradigm to identify the links between subcategories. By applying constant comparison methods, comparisons were made repeatedly to affirm the link and connections between open and axial coding categories before commencing selective coding. During the selective coding stage, the researchers identified the core category and conceptually related all categories to the core category to construct a meaningful and coherent story. This process of analysis fostered the construction of a meaningful and articulate story regarding participants’ perceptions of their parents’ roles in their career development (Strauss and Corbin, 1990; Birks and Mills, 2015). PAT and BSMA independently identified and collaboratively confirmed the categories that emerged from the data through continuous discussions.

The trustworthiness of the outcomes of the study was ensured through the four qualitative criteria of credibility, transferability, dependability and confirmability (Lincoln and Guba, 1985; Birks and Mills, 2015). Credibility of the study was achieved through data saturation. Transferability was demonstrated through purposive sampling and in-depth descriptions of the data analysis process. Dependability was ensured through field notes and memo writing that highlighted significant issues for further exploration. Confirmability was achieved through participants’ validation of the study findings for accuracy and resonance with their experiences (Lincoln and Guba, 1985). Furthermore, reflexivity was heightened by the involvement of all the researchers in the processes of data interpretation (Patton, 1999). Collecting data from several participants from different backgrounds with dissimilar characteristics (gender, entry status, level of education and duration of residence) offered dynamic perceptions on the phenomenon under consideration, which facilitated the attainment of theoretical data saturation. The analysis of the data followed concurrent data gathering until thematic data saturation was reached. Relevant exemplar responses from participants were quoted verbatim to support the results.

## Results

### Participants’ Characteristics

Thirty-one SSA migrant youths from 21 families participated in the interviews. The study participants came from eight SSA countries: nine from Congo, eight from Nigeria, seven from Zimbabwe, three from Kenya and singular participants from Eritrea, Rwanda, Sierra Leone and Uganda. These SSA communities are part of the most represented African groups in Townsville. The participants comprised 12 males (M) and 19 females (F) with length of residency in Australia ranging from one to 17 years. There were 20 high school students with an age range of 14–18 years and 11 tertiary education students whose ages ranged from 19 to 29 years. Twenty-one participants identified their residential statuses as being Australian citizens and 10 as permanent residents. Twelve participants indicated that their family had humanitarian entry (HM) status and 19 had professional entry (PM) status. Table 1 indicates the participants’ profiles.

**Table 1:** Study participants’ profile.

Interview ID (# per family)	Participants’ pseudonyms (Gender)	Level of education		Entry status	Length of residency
		Secondary	Tertiary		
1	Gideon (M)	1	–	Humanitarian	8 years
2	Daniella (F)	1	–	Humanitarian	1 year
3	Seth (M)	–	1	Humanitarian	6 years
4	Isaac (M)	–	1	Humanitarian	2 years
5	Edna (F)	2	–	Professional	17 years
	Cecilia (F)				
6	Philip (M)	2	–	Humanitarian	4 years
	Zoe (F)				
7	John (M)	–	1	Humanitarian	5 years
8	Madison (F)	2	–	Professional	12 years
	Rita (F)				
9	Naomi (F)	1	–	Professional	12 years
10	Evans (M)	1	1	Professional	6 years
	Paul (M)				
11	Angela (F)	1	–	Professional	11 years
12	Patrick (M)	1	1	Humanitarian	4 years
	Tom (M)				
13	Madonna (F)	1	1	Humanitarian	8 years
	Felicia (F)				
14	Jessica (F)	1	–	Professional	13 years
15	Eric (M)	1	–	Humanitarian	6 years
16	Gail (F)	2	–	Professional	6 years
	Charles (M)				
17	Cathy (F)	1	1	Professional	6 years
	Martin (M)				
18	Caroline (F)	–	1	Professional	7 years
19	Patricia (F)	–	1	Professional	13 years
20	Eugenia (F)	–	1	Professional	31 years
21	Denise (F)	2	1	Professional	8 years
	Nadine (F)				
	Louisa (F)				

### Perceived Parental Roles

**Figure 1:**
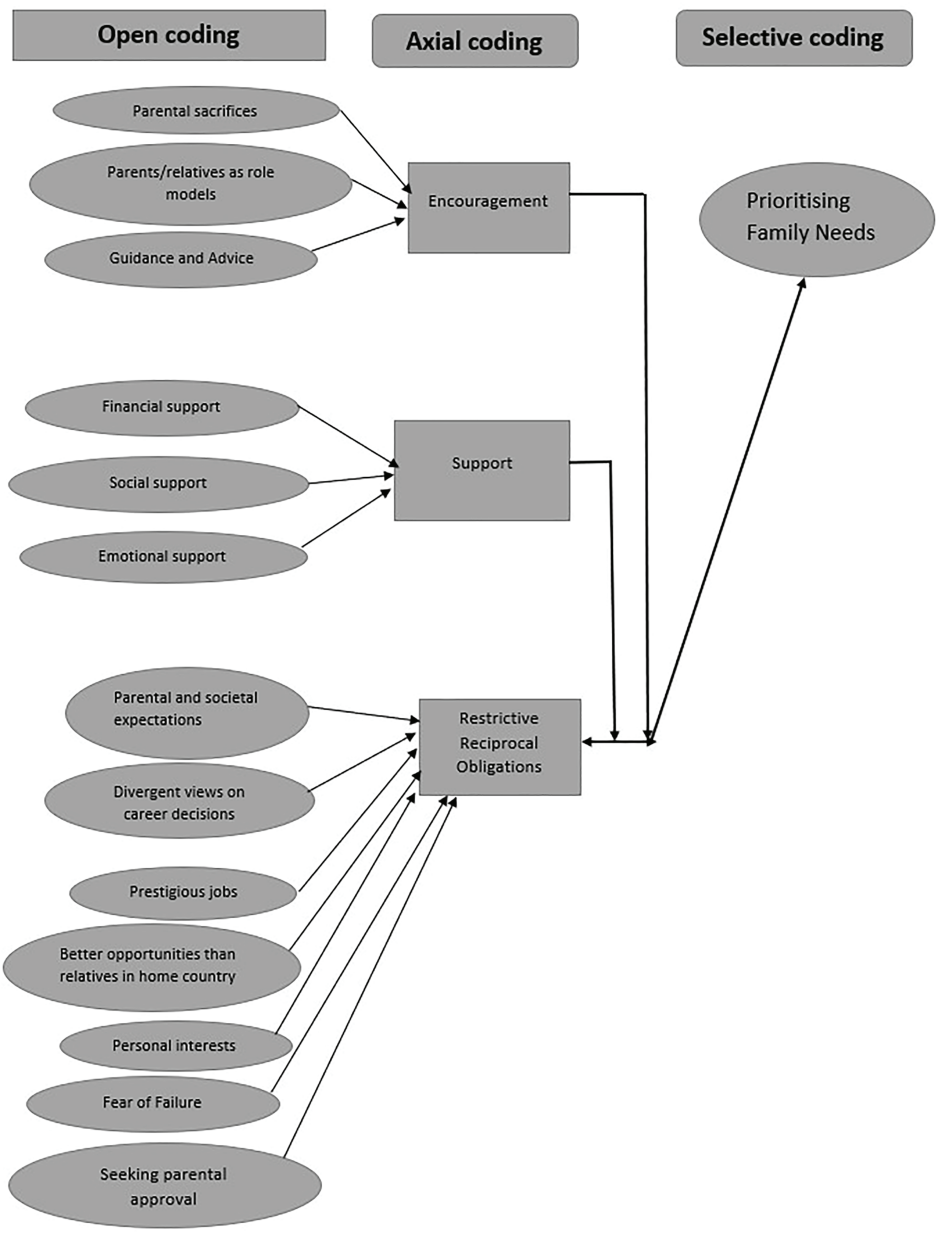
The three phases of coding.

**Figure 2:**
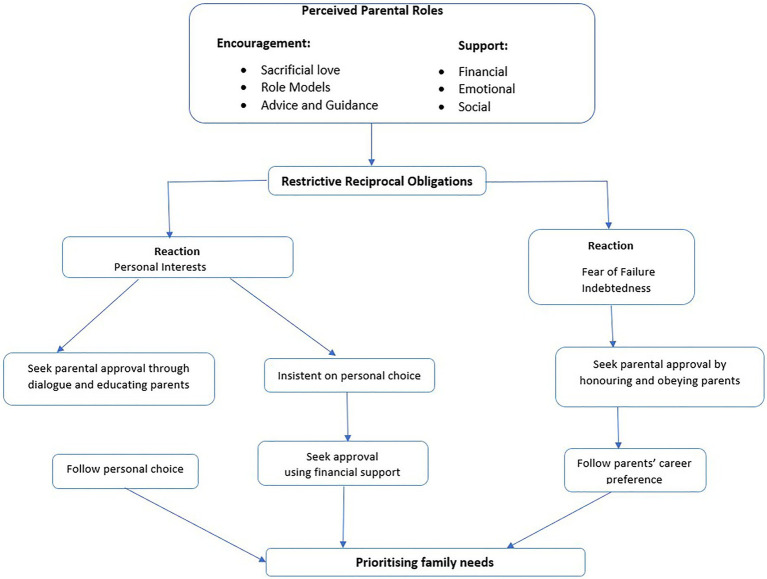
Model of sub-Saharan African (SSA) migrant youths’ perceptions of parental roles in their career decision making processes.

Figure 1 presents the results of the open, axial and selective coding. The open coding phase involved conceptual labelling and assignment of codes to chunks of data. Identified codes were clustered into 14 major codes. For the axial coding phase, open codes were grouped into categories with relationships between categories identified. Overlapping categories were refined. The cycle was repeated until theoretical saturation was achieved. Three main categories were identified, namely, Encouragement, Support and Restrictive Reciprocal Obligations. In the selective coding phase, the three categories were used to form the theory “Prioritizing Family Needs” in career decision-making, which explained the phenomenon of investigation. Most participants in this study appreciated their parents’ effort to give them a good education and a better future. As shown in Figure 2, the participants reported two major parental roles – encouragement (sacrificial love, role modeling and advice/guidance) and support (financial, emotional and social) that aided their career decision-making process. These roles were related to deeply held perceptions of parental caring, supportive and nurturing responsibilities.

#### Sacrificial Love

Most participants began with an appreciation of the sacrifices their parents made on their behalf in migrating to Australia. They also acknowledged the relentless effort of their parents to support them in their academic endeavors.

*“Family support has helped me along my career journey” (Patrick, HM, M).*

*“I think my parents and my family they were very supportive of my education, and especially considering where I come from” (Zoe, HM, F).*

*“You can’t really quantify their support for what they’ve done, and I guess also something I’d like to stress is just how they put themselves aside for us and have spent money on us” (Cathy, PM, F).*

Irrespective of their entry status or level of education, participants perceived that their parents were committed to ensure they attained the best educational and career outcomes.

*“…you should not walk this path in discovering who you are and who you’re meant to be by yourself and for society without the influence of your parents. I feel that is very important as they only have your best interest at heart” (Rita, PM, F).*

#### Parents as Role Models

Most participants in this study perceived that their parents were people to look up to *“because parents show to their children that they are there for them, not just there to tell them what to do but to show them how to do it” (Angela, PM, F).* Whether they are tertiary or secondary students, participants from professional migrant homes relied on their parents’ professional lives to figure out their career pathways and tapped into their parents’ expertise.

*“…dad’s done business and mum’s done law as well. …because they always have the experience, that’s how I look at it. So, I might sort of realise that I want to do something else but at the same time I sort of rely on them to have their input, because they’ve obviously had experience. So yeah, I would sort of choose my own way, but I need their advice as well” (Martin, PM, M).*

*“I see them both as successful professionals, so it makes sense in my head because I’ve seen it. If they weren’t providing an example for me to see it would be hard for me to believe but I would have a lot of talks, like frequent talks, [with them] on career choice and stuff” (Caroline, PM, F).*

#### Advice and Guidance

Participants also consulted their parents for advice and guidance regarding their career choice because they felt their parents know what kind of job they are best suited for, having lived with them all their lives. However, the younger children (mostly in secondary school) were more inclined to consult their parents for career guidance and they tended to follow their parents’ career preferences.

*“It’s nice to go to them when we have our ideas about our career so that they can direct us and tell us if they think we are going in the right direction or not” (Edna, PM, F).*

*“I’m really happy with my parents’ encouragement and assistance for my education and my training for future work” (Zoe, HM, F).*

Some students indicated that their fathers provided practical advice, while the mothers were more affective.

*“…like my dad, the advice he would give it’s very practical. Like this is life you know, what I mean? You can’t avoid these things. You need to go through them, and you need to find ways to deal with them. There’s no point crying because you’re going to experience worse things. So, if you can’t cope with it now, then what are you going to do later? Then in terms of my mother, it’s more of comfort and caring… So, I think in different ways their advice just provides the perfect balance of practical advice and affection” (Caroline, PM, F).*

Intriguingly, one participant highlighted an important aspect of his career decision process in which he felt his parents helped him gain a perspective of the limited options he had in his home country as against the wide range of options that are available in his new country of residence. Given this reality, he is guided to frame his career options taking these backgrounds into account.

*“Back home you don’t have any options, but the societal expectations are great. So for them, it wasn’t like what you are going to do. It is like what you are going to get from what you’re going to do, you know” (Patrick, HM, M).*

#### Financial Support

Participants from both professional and humanitarian migrant backgrounds appreciated the financial support they received from their parents and acknowledged it was vital to enhancing their academic and career outcomes.

*“I don’t even think you can quantify it, to be honest.…they absolutely exceed whatever scale there is financially, emotionally and spiritually they’ve been there the whole way” (Cathy, PM, F).*

*“…Yeah, I’m happy for their support because the biggest support is that I am in school still learning, and dad even gave a laptop to support my studies” (Eric, HM, M).*

All participants in this study affirmed that their parents provided accommodation during their studies and they were not required to pay rent. Even participants who were undertaking tertiary education received full financial assistance from their parents and relied on them for sustenance, so that they could focus on their tertiary education.

*“I still live at home so that aspect of having - not having to worry about paying rent. I don’t pay any bills, I don’t pay my - any kind of bills, my car, nothing. I don’t have to exert extra stress on myself. I think that helps because then that means I don’t have to do part-time job but - I used to work, I think, four shifts a week and my parents were like, no. They made me cut to once a week because of school” (Caroline, PM, F).*

*“I am here with my parents. I live with them and they provide everything. Maybe when I’ve got a job, that’s when I’ll move – but now, no!” (Isaac, HM, M).*

#### Emotional and Social Support

Participants turn to their parents for emotional and social support when needed. Many participants affirmed that their stability and the focus on their career paths were contingent on the support they received from their family. They depended on support from their parents to sustain their momentum in their career development.

*“If it wasn’t for my parents support and encouragement my career trajectories would be different” (Madonna, HM, F).*

*“…Emotionally and mentally there are a lot of moments where you’re sort of like really down. You’re like maybe this is not me. I want to leave this course. …With my parents, I feel like they’re obviously a huge influence in my life. So just explaining to me where I’m at, how I feel, they’re really encouraging and always saying this is right, you’ve made the right choice and you just believe in yourself - it’s part of life” (Caroline, PM, F).*

Participants who were working and studying at the same time appreciated the domestic support their families provided.

*“My family, they support me a lot in terms of cooking food - because they see me always with my books and, they really help me to overcome some of the study difficulties that really helped me to get through with my studies” (Patrick, HM, M).*

### Restrictive Reciprocal Obligations

Interestingly, in unpacking the parental roles, participants also identified familial needs and societal expectations, which presented restrictive obligations to the participants. From an early age, participants learnt from their parents about the societal values of their heritage country as their parents embody the customs and practices of their heritage societies. Therefore, to the SSA migrant youths, following their parents’ career preferences represents the fulfillment of their obligation to their family and societal expectations. In their home countries, it is the duty of the parents to provide for the children, while they are young, including their educational needs. In a similar way, the youths are obligated to reciprocate the care and support their parents had provided when the youths become adults. All participants in this study indicated that the support and encouragement they received from their parents are over and above expectation. In acknowledging their parents’ supportive role, the youths realize that their obligations become restrictive and they felt compelled to honor their parents by making career choices that made their parents proud and helped to fulfill their parents’ migration goals for the family.

These restrictive obligations presented a fork in the career decision-making road of participants (Figure 2). The participants reported that when choosing a career, they must balance their personal interests with what is acceptable to their parents. They find themselves having to make a decision on whether to conform to the career preferences of their parents or follow their own personal interests. All participants indicated that their parents had expressed career preferences for them. These participants realized that their freedom was restricted by their parents unlike their local peers.

*“…At a glance, when I look at my friends, I kind of didn’t understand how come their parents allowed them to do whatever they wanted and followed their dreams. However, when I say that it’s like, yes, you can do that as well, you can be like your friends but just remember where you come from and the expectations placed on you. You don’t want to regret it one day and say, oh, I wish I’d gone to school and done this” (Caroline, PM, F).*

*“I’ve come to this realisation that sometimes, you know, we’re tempted because you’re still in those years when your friends influence you as well. So, you’re just – you get those feelings like I should be able to use my phone as my friends but sometimes you just have to come back and remind yourself why your parents do it for you” (Rita, PM, F).*

Although the participants did not like the restrictive obligations posed by their parents, obtaining parental approval was of utmost importance to them. However, their responses to the situation and how (strategies utilized) and when (timing) they negotiated parental approval were largely determined by their migrant entry status. Majority of the PM participants, in seeking parental approval, chose to obey, honor and accept their parent’s career preferences.

*“I’m kind of used to being reassured to go the way my parents want me to go. I am trying to follow on from my parents’ profession, maybe something in nursing” (Naomi, PM, F).*

*“…my parents made me become a lawyer and now I enjoy it but when I was a little kid I wanted to become a singer, something else” (Evans, PM, M).*

*“…I think we’re lucky because our parents want it as bad for us as we want it for ourselves. So, it’s kind of like we’re in the same boat” (Rita, PM, F).*

They felt they needed to honor their parents by making choices that pleased and fulfilled their parents’ desires and made them proud.

*“I think it’s a little bit too late for me to say I no longer want to do this; I’m going to give my parents a heart attack. I feel like, okay, as much as my parents want me to do it, it’s my life and this is my future. It’s not just I’m doing it for myself, I’m doing it for my parents as well… [also] the pride that they have: like whenever the family is over in Africa, my parents tell everybody, that’s [Caroline], she’s … studying medicine. It really brings me joy. So that’s what makes it even sweeter. I feel like it’s a way of me saying thank you for where my parents have brought me thus far” (Caroline, PM, F).*

They conceded, became obedient and conformed to their parents’ career choice for fear of failure if they attempt to follow their own career pursuits. Being unsuccessful in their educational or career pursuits was considered a failure, not only by the individual but also by the family and their society.

*“I feel like if I had gone into the sport part and ignored their [the parents] advice earlier on, I don’t think if anything had happened, like say I got injured, I don’t think I would have had that same support……. It would be more like I told you so!” (Caroline, PM, F).*

This group of participants also felt indebted because of the sacrifices their parents have made for them.

*“I don’t think we can ever pay them [parents] back. But I guess after Uni and whatnot we can sort of try and – you know, also look after them as they get older” (Martin, PM, M).*

For some participants, comparing themselves to relatives still living in the heritage country helped them to appreciate what their parents have done for them.

*“I’ve seen my family members [in the home country]; they’re probably smarter than I am but it’s just because of lack of opportunity…like maybe there’s no jobs…so you see them selling things at markets. Some of them are very educated but they don’t have the chance to go to school… So, I think lack of opportunity makes all the difference” (Cathy, PM, F).*

Participants who chose to follow their personal career choices were mostly males from HM homes.

*“…My family, they wanted me to do health. They wanted me to go into health professions but I told them that, no, I don’t like that profession, there are opportunities and a lot of jobs in the accounting side so I’m going to do my accounting, so that was my decision” (Patrick, HM, M).*

*“I like soccer because we get to like travel and see around the world, [internationals]. Well, because I usually play soccer with my friends at the park in the school and the teachers as well at school” (Tom, HM, M).*

*“…as much as my parents want me to do this course. That’s my life. So, I’m the one who’s going to be working. It’s like, this is my future” (Gideon, HM, M).*

Only two female participants from PM homes also chose to follow their personal interest.

*“…I think every parent seems to think they know what’s best for you…. I think they’ve mentioned it since I left high school. They [the parents] wanted me to do nursing… I have never even thought about getting into nursing… it just wasn’t for me…. right now I am doing criminal psychology” (Patricia, PM, F).*

Interestingly, participants who stuck to their personal career choices still ensured that they gained parental approval before proceeding and the strategy they adopted in gaining parental consent was constant dialoguing with and educating parents. This group of participants were mostly the older ones who had broadened knowledge base and increased level of social capital which made them better equipped to discuss maturely with their parents if they had different career interests from what their parents preferred.

*“That’s always driven me, yeah. But I’ve also known when I grew up and I told mum, Mama, I think I want to be a lawyer when I grow up. She told me as you are a minority you have to also think that the clients you may get you will have to focus on that they might not always be the best for you are a minority. The dominant culture of the area would want to go with their lawyer, like the same as theirs. So I’ve known that the profession I want, I have to work harder. If I want to get work, I have to do over and above. I enjoy performing well in school and I enjoyed learning and stuff. So I don’t think it was, you know, much of a push or much a, you know l tried to explain to them that l was on certain direction. It just naturally happened” (Jessica, PM, F).*

*“…you have to educate them [the parents] to understand why you are directing them in this pathway… and you must be someone who is flexible to understand their views too. I had to educate them before I even made my career decision. …otherwise you can take a decision and you upset them even if it is a good decision… later they will come to understand that you did not take a decision which was bad or a decision which could take you in a blind career pathway” (Patrick, HM, M).*

Only one HM participant blatantly refused their parents’ career preference, and this caused an interim rift in the family. Nonetheless, this participant who had completed his first degree and was doing a postgraduate program at the time of this study endeavored to seek parental approval later by providing financial support to the family after commencing work.

*“…My parents sent me to school, thinking I will do what they wanted me to do. They wished I became a teacher like one of my aunts… that was the path they wanted me to take! But it was not my passion. There was a misunderstanding between my parents and myself when I chose to go into nursing! They told me you should do education! I told them mum, dad, I can’t do that! I am going to do what I want. …this misunderstanding lasted for years, until when I started working and earned good money. Then they said…that’s good!” (Seth, HM, M).*

One HM participant preferred to seek advice from their friend and teacher. However, the advice provided stirred them away from their personal career interest.

*“… when I came here, I tried to ask friends: how do you feel about my career? They told me it takes longer. I tried asking the teacher… he told me that it will take me a long way for me to become a doctor, like maybe seven years, eight years. …it’s actually a long way for me to become what I wanted to be…too long…plus it is really hard….I am just studying social work. I think it’s pretty easy for me, so I don’t want to pursue a higher career that’s going to take me too long to graduate” (Isaac, HM. M).*

All participants perceived they had an obligation to pay back their parents and the home society that nurtured them. Regardless of participants’ characteristics, they all perceived that their career choice is expected to support their family. Due to emphasis on familial/societal duties and obligations, the participants expressed their intention to return to their heritage countries after completing their education and training, so that they can support their family and community.

*“It’s not what you are going to do, it’s what you are going to get from what you do that will support your family” (Seth, HM, M).*

*“I think there’s that huge part of me, which makes me think that I owe my heart to my home country…one day I would like to go back, because that’s where it all started, otherwise I wouldn’t be here. If I hadn’t had the experience I had from my culture, I wouldn’t be where I am today” (Caroline, PM, F).*

## Discussion

The current study makes worthwhile contributions to the body of literature on SSA youths’ understanding of their parents’ role in the course of their career construction in Australia. One of the main reasons why people migrant is a desire to provide better educational and economic opportunities for their families (Jung, 2015; Lundy and Lartey, 2017). This sentiment is applicable to all our study participants regardless of their entry status, educational level or country of origin. The study participants acknowledged prioritization of education by their parents, who believed that academic achievement and occupational success are prerequisites for successful integration. These findings are in line with recent work by Albertini et al. (2019) who posited that education is an investment for youths’ future occupational success which also benefits the entire household’s economic wellbeing.

Overall, our findings suggest that all participants had a positive perception of their parents’ roles. The participants identified their parents’ roles as providing support (financial, emotional and social) and encouragement (sacrificial love, role modeling and advice/guidance). These findings are in line with earlier studies, which documented that youths perceive their parents as being their most influential role models (Brown and Treviño, 2014), and sources of motivation and wisdom (Karunanayake and Nauta, 2004). Participants in this study tended to turn to their mothers for solace and assurance, while their fathers challenged them to persevere in times of difficulty. The findings of this study align with earlier research on the patterns of advice provided by mothers and fathers (Germeijs and Verschueren, 2009). According to Soenens and Vansteenkiste (2005), fathers tend to challenge their children to aspire towards greater heights but they may not be expressive on an emotional level. Mothers, on the other hand, tend to be more comforting and friendly (Germeijs and Verschueren, 2009). The high school students relied on the guidance of their parents when making important decisions, while the tertiary students sought encouragement and support to maintain momentum while navigating their career trajectories. Previous studies have found that younger adolescents perceive more support from their parents, while older ones are more inclined to perceptions of parental coercive control (Degoede et al., 2009). Most of the participants who turned to their parents during the times they felt academically stressed and down spirited were from PM backgrounds, while only three HM participants sought parental advice during stressful academic periods. These numbers reflect that the students from PM backgrounds were more likely to consult with their parents for career advice because they perceived them as role models who have the knowledge required to provide the much needed career guidance.

Another factor that needs further pondering is the career histories of the parents and their education backgrounds. The reason is that these form the first and immediate career contexts the participants were exposed to and might influence the participants’ career constructions. In their narratives, both Martin and Caroline drew attention to the career affordances their parents created and this indicates that the parents were not only role models, they also produced career backgrounds from which the children could start their own career explorations. Parents who entered as professional migrants had higher levels of education and qualifications with enhanced employability in Australia as they had jobs already lined-up for them before entry into the country. As these people had migrated voluntarily, it is an indication of their competitiveness in the labour market; therefore, they are more confident and upfront with their offspring regarding their career directions. However, the humanitarian migrants who have been forced to relocate due to troubles in their home countries could have been limited in their ability to influence their children’s career pathways. As indicated by some participants and in our previous study (Akosah-Twumasi et al., 2020b), some of the humanitarian migrants with lower educational and socio-economic backgrounds may not have the confidence or the necessary resources to provide the required career guidance to their children. Parents with higher education and workforce skills, who were forced to migrate as humanitarians to escape persecution, had the educational acumen and career know-how to guide their children’s career pathways. However, as these parents may have been unable to prepare ahead of their settlement into the host country, they possibly experienced delays in gaining employment that could give them the social status and financial power to be more assertive about their children’s career decisions. It is worth noting that the varied lengths of residency of the participants did not influence their career outlooks, and this is probably because their career experiences were majorly determined by the entry status and educational backgrounds of the parents.

Often, the parents’ deep love for their children inspired them to go beyond their call of duty. However, the parents’ commitment to duty in providing the needs of their children ultimately made them feel that they were entitled to have a say in their children's career development (Akosah-Twumasi et al., 2020b). This finding is supported by the works of Lowe et al. (2015) who argued that if parents provide financial support to fund their children’s education, they are entitled to participate in the decision processes of their children’s education and career selection. From the perceptions of the study participants, their parents’ commitment to duty of care became restrictive. As a result, the participants had feelings of indebtedness and restrictive reciprocal obligations to their parents/families. Participants’ familial experience and expectations engraved in their impressionable minds that their career choices must align with their parents’ preferences and decisions must be made not only for their own sake but also for the collective good of the entire family (Sawitri et al., 2014; Kim et al., 2016).

In-depth exploration of the findings revealed that participants’ career choices are filtered through the lens of the SSA’s acculturation model – prioritizing family needs and societal expectations (Akosah-Twumasi et al., 2020a). These familial and societal expectations serve as restrictive obligations that present a “fork in the career decision-making road” – a dilemma as to which career option to choose. This confirms that youths of collectivist orientations tend to pursue group/societal goals and not their personal interests, emphasizing the standards and importance of relatedness and family cohesion (Kim et al., 2016; Akosah-Twumasi et al., 2018). The current study outcomes lend support to a recent finding by Albertini et al. (2019), which suggests that intergenerational support is pivotal for migrant youths’ socio-economic integration into the host society. The phenomenon of intergenerational support is a standard practice in many collectivist societies. For example, Filipinos regard living together with relatives, as a coping mechanism to cater for the needs of family members (Hofstede, 2001). It is therefore the norm for adult offspring to provide financial support to parents in their older age (Hofstede, 2001). Family solidarity and commitment are also the normative practice of Chinese families as they maintain filial obligations to provide help to elderly parents (Ofstedal et al., 2004; Thang, 2010; Guo et al., 2012). Whether they live in their home country or they have migrated, families from SSA and Asia tend to maintain their heritage societal practices of familial duty and reciprocal obligations to the community (Sue and Okazaki, 1990; Kao and Thompson, 2003; Zhang, 2004; Akosah-Twumasi et al., 2020a).

A sense of indebtedness to parents and society is embedded in SSA parent–child relationships, and children are expected to follow through due to cultural practices and as a way of repaying their parents for their sacrificial love. These findings are consistent with the assertion by Ma et al. (2014) that Asian migrant children’s sense of indebtedness is grounded in their cultural orientation as their response to the sacrifices parents made during their migration journey. The responsibility of restrictive reciprocal obligations compelled some participants to conform to parents’ career choice because of repercussions or the backlash of fear of failure (Wambu et al., 2017). The implication of this line of action is that SSA youths may struggle not only in the pursuit of their career training but also with their work-life balance in the future, which may affect their health, social life and psychological wellbeing (Laursen and Collins, 2009). There are potential consequences for the participants who conform to their parents’ career preferences. If the career choice favored by their parents leads participants to a fulfilling career path that they enjoy, they would forever be grateful to their parents. On the contrary, if participants do not enjoy their job and feel unfulfilled by the type of profession chosen for them, this could affect their psychological connectedness with their parents, which may erode participants’ loyalty and obligation towards parents for making them follow a career path that they despise (Gavazzi et al., 1999).

PM participants who followed their personal interests for the most part dialogued with their parents, while the HM participants utilized strategies, such as educating parents and providing financial support after commencing work to obtain parental approval. In our previous study, trusting parents reported having limited understanding of the education system and a lack of confidence in their ability to provide their children with guidance on career choices (Akosah-Twumasi et al., 2020b). In this case, educating parents was an effective strategy to enhance the parents’ knowledge of the employment prospects of their children’s chosen career in Australia. In some circumstances, participants consulted other significant figures, like friends and teachers, on career related matters. Isaac [HM, M] demonstrated this by conferring with his educators and peers about his desire to become a doctor and the required years of training. This finding is consistent with recent research by Bartoszuk et al. (2019) who postulated that if students perceive that the level of support they are getting from parents is insufficient, they may look elsewhere for the support they need. This implies that some humanitarian migrant students may rely on friends and teachers for career guidance instead of their parents. Peer and educator influence could change the dynamics of the parent–child relationship, and this may warrant future exploratory studies.

The participants who opposed their parents’ career choice risked facing dire consequences as their refusal to follow their parents’ career directions resulted in a rift in the family. This finding is consistent with earlier research by Ma et al. (2014) who contended that career decision-making conflicts between parents and children of Asian descent brought a sense of guilt to their children leading to possible repercussions with their health and wellbeing. As evidenced in this study, rifts occurred within some families when some participants insisted on pursuing their personal interests. This study outcome aligns with earlier research by Rao and Walton (2004) who reported that any opposition to the collectivist family’s career preference and the desire to pursue one’s personal interests could be construed as disobedience, warranting familial/societal sanctions. Nonetheless, they still kept their psychological connectedness with their parents, and eventually, such rifts were repaired when participants negotiated with their parents after they had secured their career and were gainfully employed. These participants must be courageous and brave because talking back at parents, especially in the African cultural context, could have serious repercussions. Ultimately, migrant SSA youths acknowledge the potential challenges posed by restrictive parental obligations. However, parental approval is essential, and whether these young people went with their parents’ choices or not there appears to be reconciliation with family in the end.

### Practical Implications

The theoretical framework identified in this study which is restrictive reciprocal obligations that compel SSA youths to prioritize their family needs and seek parental approval when making career choices aligns with the SLTCDM which posits that socio-cognitive beliefs are influenced by various factors which could facilitate or inhibit the career decision-making behavior of individuals (Krumboltz, 1994). In this study, such factors include environmental conditions and associative learning experiences, in which socio-economic and cultural sources influence skill development, interpretations of self-observations and world-view generalizations (Krumboltz and Nichols, 1990). This theory provides in-depth understanding of the career development trajectories of youths from collectivist societies as it highlights the influential role of SSA parents in ensuring that their children prioritize family needs in their career choice decisions. This implies that for this group of people, career decision-making processes may not be internalized within the individual youth’s mind but is rather processed by the family unit and transmitted through social learning to the young adult. Lent et al.’s (1994) SCCT model of career choice process provides further insights into the study findings. SCCT accentuates that career choice goals are sometimes less influenced by personal interest. In such cases, supportive environmental conditions may be lacking (Lent et al., 2002). The role of personal interests in career choice may be limited by self-efficacy beliefs, outcome expectations, cultural values or environmental variables. In such instances, people may need to compromise their interests and, instead, make their choices on the basis of pragmatic considerations, such as full support of important others, perceived ability, job opportunities/accessibility and financial remuneration (Lent et al., 2000, 2002).

Data from this study suggest that parental influence based on cultural beliefs/values and outcomes expectations could pose as barriers, preventing youths from taking control of their lives and exploring career opportunities that align with their personal interests. In this study, we note that career decision-making is not a personal individual process for the SSA youth. However, the significant and highly influential role of parents in the career trajectory of young people from collectivist societies is not quite explicit in existing career theories which are more closely linked to individualistic cultures (Arulmani et al., 2003; Polenova et al., 2018). The findings of this study can be used to inform and develop culturally sensitive career counseling services. Career counseling that addresses career beliefs, values and goals could create a platform upon which migrants from collectivist societies approach career decision-making. Career counseling with SSA migrant clients requires understanding of their unique and complex circumstances, and a set of practical tools specifically tailored to address issues of migrant career development (Chen, 2009). An essential role of counselors is to assist students with establishing rapport with their parents on career matters. School career counselors could re-orientate SSA migrant parents by offering them courses and training programs to broaden their resource base on available career options (Ma and Yeh, 2010). In doing so, the career counselors can dispel the erroneous impressions associated with fear and shame connected with lower academic aspirations and failure. Replacing the failure mentality with resilience, confidence and a "can do" spirit will assist youths in identifying the abilities and skills that are best suited to their career interests (Wambu et al., 2017).

The study findings also call attention to the intergenerational differences between the career schema of the parents and the children, which sometimes create tensions and conflicts. Such tensions and conflicts stem from perceptions of generational differences in values, behavior and/or identity (Foster, 2013). The children may be operating from another set of schema mainly based on career information available in their new contexts, particularly in the light of constant advancement of technology which brings about new experiences that provide opportunities for them to discover new interests. The difference of values, behavior and goals between generations may negatively impact on self-efficacy beliefs (Lent et al., 1994, 2002), and this needs to be considered by career counselors with the development of strategies that would help SSA clients identify their personal interests and work values. School counselors and other service providers could further assist SSA migrant students with culturally responsive programmes to increase the participants’ social capital, which will facilitate and support their career decisions processes. Given that SSA migrants are used to consulting significant others when making major life decisions, shadowing a mentor during their career decision trajectories for instance, will be beneficial to them.

### Strengths and Limitations

The major strength of this study is the use of an exploratory research design to unpack the complexity of career decision-making among SSA migrant youths and their perceptions of their parents’ roles in the process, which extends the literature on this subject. However, the study is limited to only migrant youths from eight purposively selected SSA countries who are residing in Townsville, Australia, which may impact on the transferability of the findings given the specificity of participants’ unique characteristics (for example, most participants were children of professional migrants). Nonetheless, the study findings may provide both educators, especially career counselors, and parents with an in-depth understanding of their roles in assisting in the career development trajectories of SSA migrant youths. Future longitudinal studies are required to explore the occupational outlooks of the participants who opposed their parents’ career choice and followed their own personal interests and those who chose to follow their parents’ career preferences for fear of failure. Such longitudinal studies could also provide insight into how these migrant youths will approach the career decision-making of their own offspring.

## Conclusion

This study has implications for teachers, career counselors and other professionals as well as for the parents of SSA migrant youths. The strong value of education buttressed by a sense of obligation to the family bodes well for the educational development of SSA migrant youths. The study findings highlight the SSA migrant youths’ sense of responsibility towards their families and society (reciprocal obligations). This calls for teamwork between their parents and counselors so that the children are better supported and guided through their career related challenges. Due to the apparent culturally non-negotiable nature of SSA migrant children’s reciprocal obligation, it is incumbent on career counselors to allay the children’s fears of potentially disappointing their parents through perceived poor performance or academic failure because they chose to pursue careers based on their personal interests. Programmes by policymakers, building on the SSA cultural traditions could provide these migrant families with the required resources to assist their children to become economically productive adults in the host country.

## Data Availability Statement

The original contributions presented in the study are included in the article/supplementary material, further inquiries can be directed to the corresponding author.

## Ethics Statement

This study was reviewed and granted ethics approval by James Cook University’s Human Research Ethics Committee (ethics approval numbers - H7006 and H7374). For participants below 18 years, written informed consent to participate in the study was provided by their legal guardian/next of kin.

## Author Contributions

PA-T and BM-A: conceptualization, data curation, formal analysis, methodology and validation. PA-T: funding acquisition, investigation and writing – original draft. BM-A: project administration and resources. BM-A, TE, DL, and KT: supervision. PA-T, TE, DL, KT, and BM-A: writing – review and editing. All authors have read and agreed to the published version of the manuscript.

### Conflict of Interest

The authors declare that the research was conducted in the absence of any commercial or financial relationships that could be construed as a potential conflict of interest.
